# Increased vertebral canal diameter measured by ultrasonography as a sign of vasculitis in patients with giant cell arteritis

**DOI:** 10.3389/fmed.2023.1283285

**Published:** 2023-11-07

**Authors:** Oscar Ayo-Martin, Jorge Garcia-Garcia, Francisco Hernandez-Fernandez, Maria Palao, Beatriz Poyatos-Herraiz, Tito Humberto Barahona-Espinal, Alberto Gonzalez-Romero, Ester Marin-Conesa, Blanca Serrano-Serrano, Maria Paya, Tomas Segura

**Affiliations:** ^1^Laboratory of Neurosonology, Department of Neurology, Complejo Hospitalario Universitario de Albacete, Albacete, Spain; ^2^Instituto de Investigación en Discapacidades Neurológicas (IDINE), Universidad de Castilla-La Mancha, Albacete, Spain; ^3^Faculty of Medicine, Universidad de Castilla-La Mancha, Albacete, Spain; ^4^Department of Neurology, Hospital Militar de Honduras and Hospital DIME, Tegucigalpa, Honduras; ^5^Department of Neurology, Hospital General Universitario Reina Sofía, Murcia, Spain

**Keywords:** giant cell arteritis, temporal arteritis, vasculitis, vertebral artery, stroke, neurosonology, ultrasound

## Abstract

**Introduction:**

The diagnosis of giant cell arteritis (GCA) by ultrasonography including large vessels, apart from the temporal artery increases the sensibility of the study and informs about the risk of specific complications. However, there is less information about the study of these arteries, whose affection carries higher proportion of severe complications.

**Objectives:**

To describe and analyze the value of the diameter of the cervical vertebral canal of the vertebral artery (VA) as a sign of vertebral vasculitis (VV) related to GCA and estimate the risk of stroke complications.

**Materials and methods:**

Observational study of a population that includes patients with GCA with and without VA vasculitis as well as healthy subjects. We evaluated whether there were differences in VA diameter in the groups and, if so, we estimated the diagnostic capacity of the variable that best defines VA diameter using a ROC curve. Cut-off points with their associated reliability chosen thereafter.

**Results:**

There were 347 subjects included:107 with GCA of whom 37 had vertebral vasculitis, 240 healthy controls. In patients with GCA and VV, the VA diameter was increased (No GCA 3.4 mm, GCA without VV 3.6 mm, GCA with VV 5.2 mm *p* < 0.01). According to the ROC curves, the variable defining vertebral diameter with best diagnostic accuracy is the sum of both sides (area under the curve of 0.98). With a cut-off point of 8.45 mm, the reliability values are: sensitivity 94.1%, specificity 94.5%, PPV 82.1% and NPV 98.4%. With a cut-off point of 9.95 mm, the sensitivity is 52.9% and the specificity is 100%. Likewise, VA diameter is independently associated with the presence of stroke in the vertebrobasilar territory (OR 1.6, range 1.2–2.2).

**Conclusion:**

The VA diameter, measured as the sum of both sides, is an objectively measurable sign with very high reliability for detect vertebral vasculitis in patients with GCA. It is proposed here as a novel echographic sign, which can aid the detection of the involvement of an artery where the complications are especially serious.

## Introduction

Giant cell arteritis (GCA) is the most frequent primary systemic vasculitis in adults (1). It is a granulomatous arteritis of large and medium-sized vessels, mainly affecting the supra-aortic trunks and their branches. Superficial temporal artery involvement is characteristic and is responsible for most of the typical symptoms associated with this disease (2, 3).

Depending on the arteries affected, GCA can cause potentially serious complications such as permanent visual loss, aortic aneurysm and dissection, ischemic stroke, and limb arterial ischemia. Even, it is infrequent the presence of vasculitis in large vessels (main branches of supraortic trunks), this subgroup of patients carries more frequently these severe complications (4).

Complications in the nervous system, mainly ischemic stroke, are mostly due to the involvement of the extradural vertebral arteries, rather than carotid and intracranial vasculitis (2, 4–6). In more than half of the cases, strokes are attributable to GCA as a result of vertebrobasilar system involvement (7). Vertebral artery (VA) involvement, which causes neurological deficits in the brainstem and/or cerebellum, results in high mortality if not diagnosed and treated in time (2, 7). In contrast, documented involvement of intracranial vessels in GCA is very rare (7).

In general, GCA is a rare cause of stroke. Stroke occurs in only 3 to 7% of cases but is the leading cause of death in patients with GCA (7, 8). Nevertheless, compared to atherosclerosis, stroke recurrence and mortality is much higher in GCA (9, 10). Although rare, it is important to identify them early on because these patients have a high mortality rate, however, treatment is available that drastically modifies this outcome if commenced promptly (11).

The classic clinical presentation of GCA revolves around cardinal signs and symptoms stemming mainly from systemic involvement and extracranial arteritis: subacute or chronic headache, constitutional syndrome, fever, elevated acute phase reactants, and macroscopic changes in the temporal artery. Indeed, due to the frequency with which they occur, the American College of Rheumatology use these as their clinical diagnostic criteria (12), currently considered the reference diagnostic benchmark for GCA and, in fact, constitute the diagnostic gold standard superseding all available complementary tests. Although these manifestations are frequent, a high percentage of patients exists where the diagnostic criteria are not met, this percentage being 27% according to a systematic review of cases (13). Indeed, the patients affected by vasculitis in large arteries often present with non-specific symptoms (4). Those uncommon symptoms can be the only presenting clinical picture. Thus, there are situations in which patients may present with a clinical picture that in most cases is very clear, allowing the diagnosis by the established diagnostic criteria; but also, others with very nonspecific symptoms in which the initial clinical suspicion may be, in some cases, low (3). According to some case series of GCA patients affecting the vertebral arteries, all patients presenting with stroke. Most of them did not have the classical symptoms of GCA associated with stroke (11). This subgroup of patients with VA involvement resulting from GCA is associated with increased mortality rates (3, 4, 7, 11). In these latter patients, the diagnosis becomes a challenge, since the detection of the disease, its treatment, and the speed of its onset and the swiftness of treatment can influence mark the appearance of complications and therefore the short and long term prognosis. For this reason, complementary tests are warranted in order to achieve rapid diagnosis with multiple diagnostic techniques having been developed to aid diagnosis promptly and in the greatest number of patients. All are based on the demonstration of vessel inflammation. Until a few years ago, the principal technique available was temporal artery biopsy. Indeed, it is still the reference technique in many centers for the diagnosis of GCA and remains as the first diagnostic test in some guidelines (14). However, it does have several disadvantages: delay in performing and obtaining the PA report and false negatives due to inadvertent biopsy of non-inflamed areas. In addition, it can be a crude technique, with potential for complications such as ischemia of the territory supplied by the artery which, importantly, includes facial skin. Due to this, alternative non-invasive techniques have been introduced, such as angioCT, angioMRI, PET, and temporal artery ultrasound (15).

Of these, it is worth noting that, in recent years, color duplex ultrasound has been proposed as a non-invasive diagnostic tool and as a screening test for suspected GCA (16, 17). The demonstration of a concentric-shaped hypoechoic area around the temporal artery (halo sign) is a common feature of GCA and is indicative of vasculitic mural edema (17, 18), which may lead to stenosis or even occlusion (altering the flow profile and changing the velocity of the blood flow in the affected areas of the vessels) (19). Meta-analyses indicate high reliability for this sign in the diagnosis of GCA (15, 17, 20–23). Since ultrasonography is an easy and accessible technique and reproducible with good reliability, it has been postulated in different guidelines as the first diagnostic test for suspected GCA (3, 17).

Furthermore, the halo sign is not exclusive to the temporal artery (19). In recent years, similar findings have been described in other arteries such as the vertebral, occipital, or axillary arteries. This points to the possibility of finding vasculitic signs by means of ultrasonography (11, 23–25).

The study of other vascular beds may allow considerably improved sensitivity of the test, and indeed this extended ultrasound protocol is recommended in multiple guidelines (3, 16, 17, 23, 26). It also allows identification of which arteries are affected and, in turn permits which specific complications the patient may suffer from to be predicted, for example, stroke in patients with vertebral involvement.

Thus, ultrasonography, which is a widely available technique allows fast and reliable diagnosis of GCA in many patients, enabling effective treatment to be started promptly thereby lessening the chances of severe complications. This benefit is not limited solely to patients who are screened for specific suspicion of GCA. There are some diseases, such as ischemic stroke, in which ultrasonography is routinely used in the acute phase of the disease. Thus, even in patients with stroke as the only manifestation of GCA, without any cardinal symptoms as described in the diagnostic criteria, this disease can be diagnosed early in those centers where the staff are familiar with the typical vasculitic signs of GCA in the vessels usually studied (supra-aortic trunks and Circle of Willis).

In the subgroup of GCA patients suffering from ischemic stroke, the VA is the most frequently artery affected (6, 7), especially in the pars vertebralis, within the vertebral canal. There are several well described ultrasound signs used to detect this vasculitis, primarily consisting of the demonstration of a concentric thickening of the wall (macaroni sign) (11, 27, 28). This is detectable in many affected patients. The physicians in charge of the Neurosonology Laboratory of our center have analyzed a large number of patients allowing us to become familiar not only with the typical halo image in the temporal artery, but also with the vasculitic findings in other arteries. As a result, it has been possible to subjectively confirm that those patients with GCA who present vasculitic involvement of the VA are accompanied by a striking thickening of the diameter of the vertebral canal along the course of the artery between the transverse processes of the vertebrae (pars vertebralis or V2 segment). To our knowledge, this finding demonstrating pathologically increased thickness, has not previously been described as a sign of VA vasculitis. In this article, we present our case series of patients with GCA, with and without VA involvement. The main objective is to analyze whether GCA with vertebral vasculitis (VV) is accompanied by a pathological increase in the diameter of the vertebral canal and whether this diameter is a reliable diagnostic sign for vasculitis in this artery. Since this sign is easy to identify, were it to be included in diagnostic criteria, there would potentially be an increase the ability to diagnose GCA, even in those patients with paucisymptomatic or less frequent but more severe presentations (stroke). In these patients the ultrasonographic study of the acute phase may be the key to reaching the etiological diagnosis early enough for effective treatment and to avoid the poor prognosis that accompanies the subgroup of patients with GCA and vasculitic involvement of the vertebral arteries.

## Patients and methods

### Study population and ultrasonographic assessment

The design of the study corresponds to a unicentric retrospective observational study carried out in the Neurology Department of the Complejo Hospitalario Universitario de Albacete. A database was created for every patient diagnosed in our center (World Health Organization International Classification of Diseases version 9, code 446.5 and version 10 codes 31.5 and 31.6) between 2012 to 2022. The diagnosis for each case was checked for compliance with the diagnostic criteria of the American College of Rheumatology (12) and confirmed according to follow-up during 6 months. From this initial list, we selected those subjects who had undergone an ultrasonographic study in the Ultrasonography Laboratory of the Neurology Department, as part of the diagnostic process before starting treatment for GCA. The ultrasonographic study was carried out with the same devices (Esaote MyLab models 70 and 9, Modena, Italy) by a neurologist specifically trained for the diagnosis of GCA, following international standards (17).

The ultrasonographic study included in all patients the analysis of the cervical trajectory of the supra-aortic trunks the arteries of the Circle of Willis, and the temporal and occipital arteries. In patients with visual symptoms, a study of orbital blood vessels was added.

For superficial TA we used an 8–24 MHz frequency transducer [B-mode frequency, 18 MHz; depth, 15 mm; focus point below the artery, depending on the depth of the segment; color Doppler frequency, 12.5 MHz, pulse repetition frequency (PRF), 1.5 KHz]. The B gain was bright enough for distinguishing lumen and halo. Colour gain was the maximum possible without covering the halo. A 8–14 frequency transducer was used for extracranial arteries (B-mode frequency, 6 MHz; depth, 3 cm; focus point under the artery studied, depending on the depth of the segment; color Doppler frequency, 3.3 MHz; PRF, 1.0–1.5 kHz). Colour box was at least 15° between artery flow and sound waves.

All retained data from these studies are stored, including an image of any artery, suitable for morphological and hemodynamic measurements.

**Figure 1:**
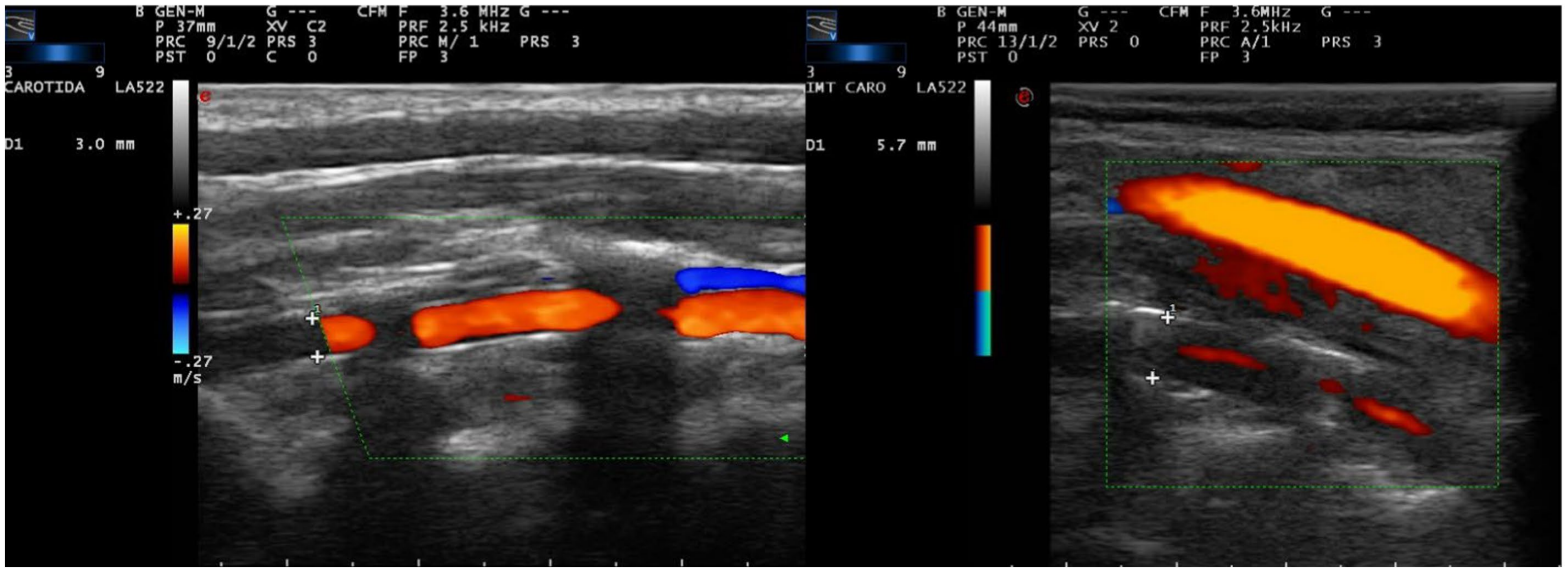
Color mode ultrasound at cervical level in paramedial sagittal projection, centered on the pars vertebralis of the vertebral artery, inside the vertebral canal. The diameter of the vertebral canal is measured defining the limits of the canal by the anterior and posterior borders of the bony surface within the vertebral canal (crosses). This value is referred in the left side of each image. **(A)** Vertebral artery of normal morphological characteristics and diameter (3.0 mm). **(B)** Common carotid artery (superficial) of normal characteristics and vertebral (deep) with data of vasculitis, with symmetric concentric wall thickening, with hypo/anechoic content, which leaves a filiform passage of central flow. The diameter of the canal is pathologically increased (5.7 mm).

The presence of cervical VA vasculitis was evaluated in the par vertebralis (V2 segment), which is the only cervical portion where wall alterations can be correctly evaluated. The ultrasonographic criteria consisted of the existence of iso or hypoechoic, concentric, homogeneous wall thickening with homogeneous content. The thickening of the wall stenoses the lumen of the artery and leaves a filiform color mode image in the blood flow with a variable degree of stenosis (Figure 1) (11, 27, 28).

Therefore, patients with GCA were divided into two groups. Patients with ultrasonographic signs of vasculitis in one or both vertebral arteries in the pars vertebralis were considered to have vertebral vasculitis. The rest were identified as patients with GCA without VV. A group of control patients without GCA, who had undergone the same ultrasonographic study, was included.

Finally, a third control group was added consisting of subjects referred to the Neurosonology Laboratory for suspected GCA in whom GCA could be ruled out. The same ultrasonographic study was performed in all cases.

The diameter of the vertebral canal along the pars vertebralis was analyzed in all study subjects from the saved images of the previously performed ultrasonographic study. All measurements were performed by the same investigator. For this purpose, the distance of the canal was calculated defining the limits of the canal by the anterior and posterior borders of the bony surface within the vertebral canal (Figure 1). Although the canal is homogeneous in diameter in most of the subjects, a minimum of 3 measurements were taken and the mean of these was calculated as the final value. Those subjects in whom the image of the VA was not appropriate for such measurement were eliminated.

The value of the VA diameter (VAD) on each side was obtained from each patient. Based on these data, two different variables were used to define the vertebral diameter. In the analytical study, the variable with the best capacity to diagnose VV was calculated. The diameter of the vertebral arteries and the vertebral canal is asymmetric in most healthy individuals. Although in most subjects the difference in diameter is small, in up to 10% of cases, the asymmetry is very marked. In these cases, one artery is hypoplastic and the other is abnormally large in diameter (27). It is considered that there is hypoplasia of a VA when the channel on one side is less than 2.0–2.5 mm or the contralateral diameter equals less than 50% of the contralateral (29). Nevertheless, the sum of both diameters remains similar to that of the general population. Therefore, a value of vertebral diameter taken individually could have less diagnostic power in diseases in which there is dilatation of the vertebral diameter. The calculation of the sum of the VA diameter of both sides may allow a better differentiation between pathological dilatations and healthy subjects with hypoplasia of one VA and contralateral compensatory hyperplasia.

Therefore, the two variables used in the analytical study were the vertebral diameter of each artery (Unilateral VAD) and the sum of the VA diameter of both sides (Sum of VAD).

### Statistical analysis

#### Baseline analysis

First, a descriptive study was carried out indicating the baseline characteristics of the sample. Categorical variables were described as percentages. Quantitative variables were indicated as mean and confidence interval (95%). The overall values and those of the three subgroups (No GCA, GCA without VV and GCA with VV) were indicated. We analyzed whether the baseline characteristics were homogeneous among the three subgroups of subjects or whether there were any variables in which the baseline values showed statistically significant differences. Since there were three subgroups, qualitative variables were analyzed using a Chi-square test, while continuous variables were analyzed using ANOVA. The results are shown numerically and graphically.

#### Analytical study

In the first part of the analytical study, we evaluated whether there was a difference in the mean vertebral diameter between the subjects of the three subgroups using an ANOVA study. The results are shown numerically and graphically. As described in the results, differences were found in one subgroup of patients (GCA with VV) versus the rest of the sample. Therefore, the sample was thereafter divided into two groups of patients: Patients without VV (No VV) and patients with VV due to GCA (GCA with VV). With the sample dichotomized into two subgroups, we analyzed by the t-Student’s test whether the mean vertebral diameter in the subjects was different between the two subgroups.

Following this, a multivariate study was performed using logistic regression to demonstrate whether the relationship between the vertebral diameter value and the presence of VV was independent of the presence of other confounding factors.

In all analyses, the vertebral diameter value was measured considering the two selected variables: Unilateral VAD and Sum of VAD.

In the second part of the analytical study, the reliability of the diagnosis of VA vasculitis in patients with GCA was evaluated using VAD values. For this purpose, a ROC curve was performed using the presence of VV (yes or no) as the dependent variable. The two indicators of vertebral diameter were evaluated as independent variables: Unilateral VAD and Sum of VAD. A ROC curve was constructed with each of the two independent variables. The ROC curve with a higher area under the curve value defined the VAD variable with the highest diagnostic capacity for the presence of VV.

From the ROC curve, the cut-off points of greatest reliability and clinical interest were selected, based on the sensitivity and specificity values they provided. The positive and negative predictive values for each VAD variable and each proposed cut-off point were calculated using the Chi-square test.

Finally, a similar analysis was performed to evaluate the association of the vertebral diameter value with the presence of GCA and with ischemic stroke in the vertebrobasilar territory. If a strong association was found, diagnostic capability was evaluated.

### Ethical-legal aspects

This study was conducted in compliance with the Declaration of Helsinki principles and received ethics approval by the local Research Ethics Committee of the Complejo Hospitalario Universitario de Albacete. No written informed consent was needed by the ethics committee because of the retrospective study design, in accordance under the national legislation and the institutional requirements.

## Results

### Study population

A total of 347 subjects were included in the study over a period from September 2021 to February 2023. Of the total sample, there were 107 (30.8%) subjects with GCA and 240 without (Table 1).

**Table 1:** Description of the sample (study population): frequency and percentage of patients.

GCA		NO GCA
107 (30.8%)		240 (69.2%)
VV	NO VV	
37 (34.6%)	70 (65.4%)	

GCA, giant cell arteritis; VV, vertebral vasculitis.

Within the group of GCA patients, 37(34.6%) had vasculitic involvement affecting the cervical segment of some VA (GCA with VV).

The mean age of the sample was 73.1 years. The distribution according to sex was 54.1% female and 45.8% male (Table 2). The ANOVA study showed that the sample was not homogeneous in age (Table 2), indicating that patients with CGA were older. In the case of sex, the Chi-square study showed no statistically significant differences (Table 2).

**Table 2:** Baseline characteristics of the whole population and subgroups.

	TOTAL	No ACG	GCA without VV	GCA with VV	*p*
Age (Years)	73.1 (11–91)	70.8	76.7	78	<0.01
Sex (Female %)	54.2	54.1	54	48.6	0.83

GCA, giant cell arteritis; VV, vertebral vasculitis.

### Stroke in vertebrobasilar territory

Of the 347 patients studied, 17 had a stroke (4.9% of the total sample, 15.9% of patients with GCA) (Table 3). All strokes affected the vertebrobasilar territory.

**Table 3:** Clinical diagnosis of stroke in patients with GCA and patients without GCA.

		Stroke	No stroke	*p*
No GCA		0 (0%)	240 (100%)	<0.01
GCA	No VV	2 (3.2%)	68 (97.1%)	
	VV	15 (38.9%)	22 (61.1%)	

GCA, giant cell arteritis; VAD, vertebral artery diameter; VV, vertebral vasculitis.

All the strokes occurred in patients with GCA. Moreover, within patients with GCA, 15 were in patients with VV. The proportion of patients with stroke was significantly higher in the subgroup of patients with signs of vasculitis in the vertebral arteries (88,25) (Table 3).

### Analytical study

#### Diagnosis of vertebral vasculitis associated to GCA

**Figure 2:**
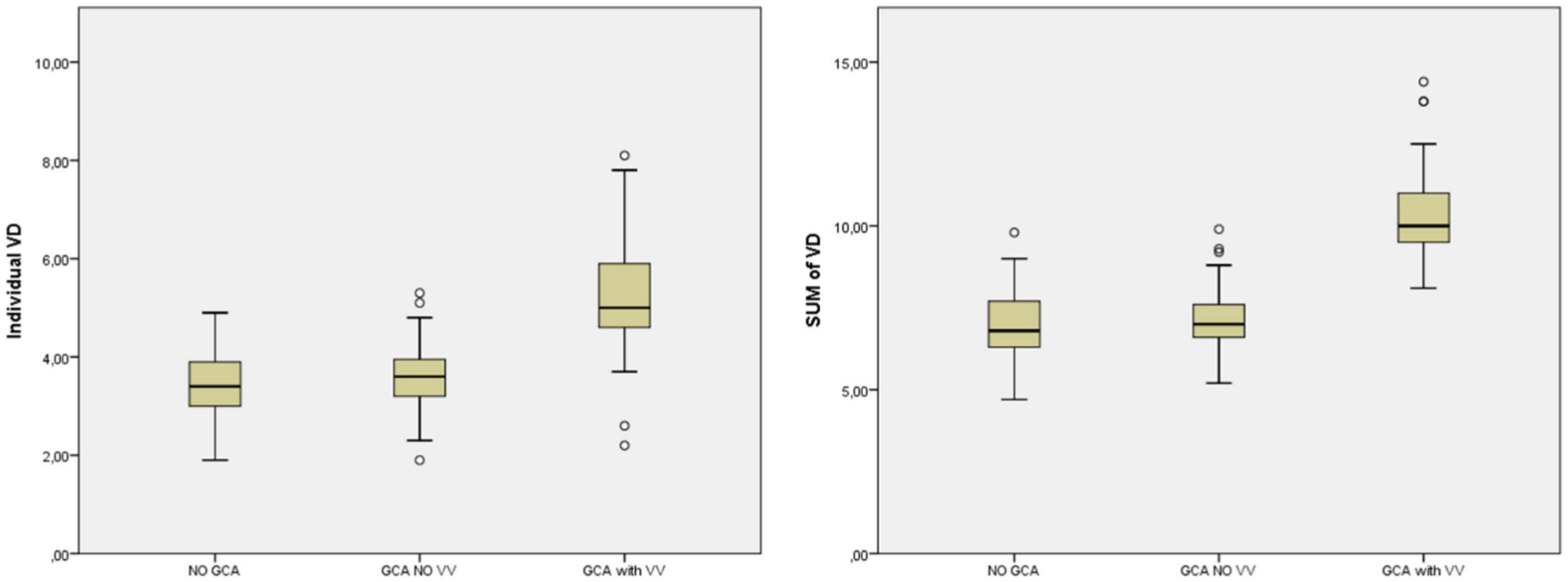
Mean VAD in patients without GCA, GCA without VV and GCA with VV. GCA, giant cell arteritis; VAD, vertebral artery diameter; VV, vertebral vasculitis.

The analysis of the VAD showed differences between the three groups (Table 4). It was demonstrated using the two variables considered (Individual VAD and Sum of VAD) for VAD. Specifically, the subgroup of GCA patients with VV showed obviously higher VAD values compared with the rest of the patients (GCA without VV and No GCA) (Figure 2 and Table 5). In a multivariate analysis, the larger VAD in the subgroup of patients with GCA with VV was independent of the confounding factors studied. With these three variables, the logistic regression model has an *R*^2^ of 0.76. The Sum of VAD reveals a statistical association (*p* < 0.01) and an OR of 8.1 (3.9–16.9). Neither age nor sex have a statistical association with the presence of VV.

**Table 4:** ANOVA test to compare the means of VAD diameter in patients without ACG, ACG without vertebral vasculitis and ACG with vertebral vasculitis.

	No GCA	GCA no VV	GCA with VV	*p*
Unilateral VAD	3.4 (3.3–3.5)	3.6 (3.5–3.7)	5.2 (5.0–5.5)	<0.01
Sum of VAD	6.9 (6.7–7.1)	7.2 (6.8–7.5)	10.4 (9.8–10.9)	<0.01

GCA, giant cell arteritis; VV, vertebral vasculitis; VAD, vertebral artery diameter.

**Table 5:** *t*-student test to compare the means of VAD diameter (mm) in patients with and without vertebral vasculitis.

	No VV	VV	*p*
Unilateral VAD (mm)	3.4 (3.3–3.5)	5.2 (5.0–5.5)	<0.01
Sum of VAD (mm)	6.9 (6.7–7.1)	10.4 (9.8–10.9)	<0.01

VV, vertebral vasculitis; VAD, vertebral artery diameter.

**Figure 3:**
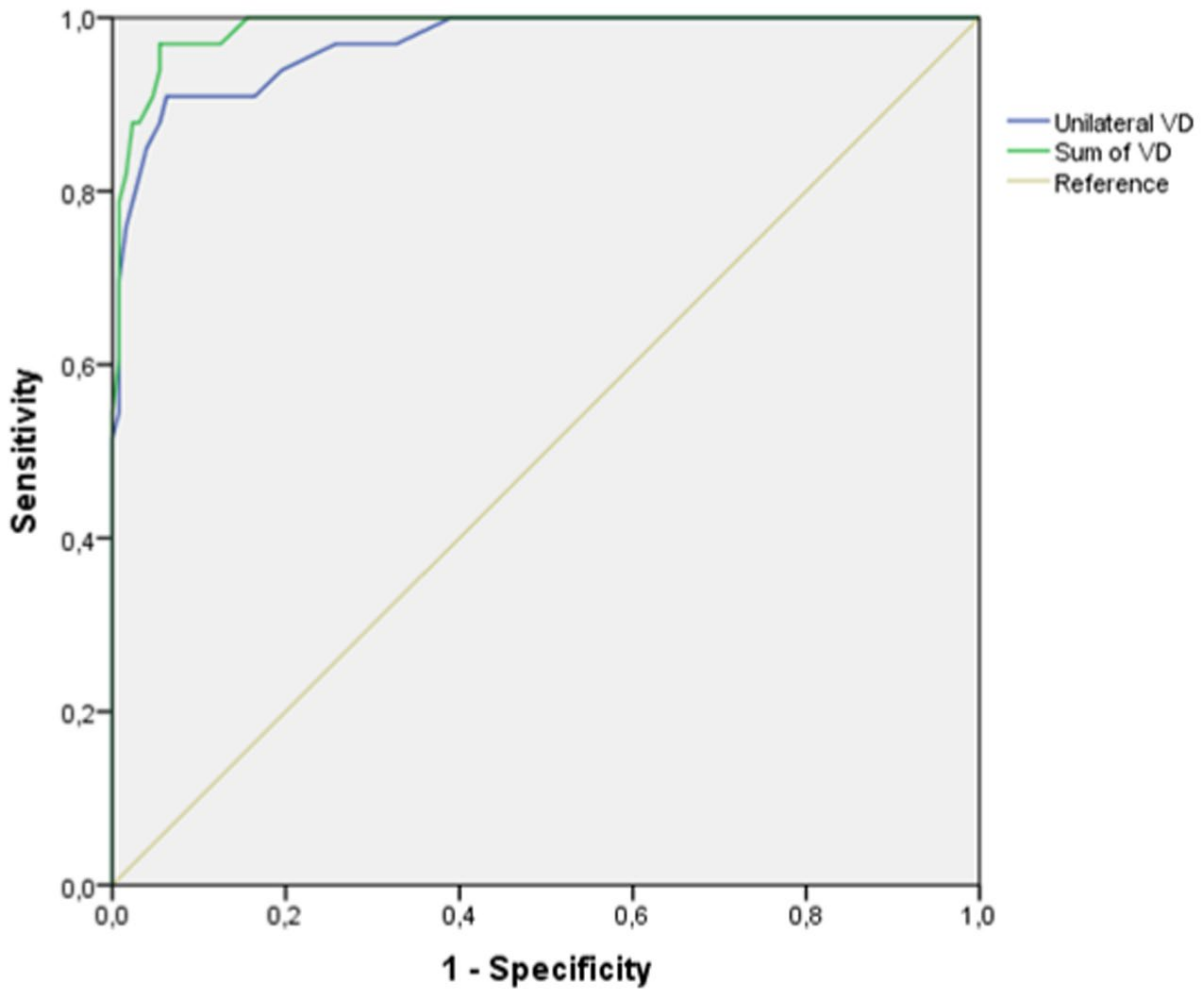
ROC curve for the evaluation of the validity of the variables of vertebral artery diameter to diagnose vertebral vasculitis due to GCA.

In the analysis of the ability to detect VV by measuring VAD, the two variables used (Unilateral VAD and Sum of VAD) show a very high area under the curve on the ROC curve (AUC Unilateral VAD: 0.96, Sum of VAD: 0.98). The variable Sum of VAD showed the highest reliability (Figure 3).

Regarding the selection of the cut-off points with the greatest diagnostic capacity, in both variables there were two values of clinical interest: one with a lower value in which the balance of sensitivity and specificity was very favorable. The other cut-off point, with a higher value, offers a specificity of 100%, so that all measurements above that value correspond to patients with VV.

In the same way as the ROC curve values, the sensitivity, specificity, positive and negative predictive value were better when using the sum of the VAD of both sides as the variable (Table 6).

**Table 6:** Cut-off points selected as the value of the variables which measures VAD: unilateral VAD Sum of VAD as discriminating cases of VV.

	Cut-off value (mm)	True/False Positives	True/false negatives	Sensitivity (%)	Specificity (%)	PPV (%)	NPV (%)
Unilateral VAD	4.35	46/25	267/6	88.5	91.4	64.8	97.8
	5.35	27/0	292/25	51.9	100	100	92.1
Sum of VAD	8.45	32/7	121/2	94.1	94.5	82.1	98.4
	9.95	18/0	128/16	52.9	100	100	88.9

PPV, positive predictive value; NPV, negative predictive value; GCA, giant cell arteritis; VAD, vertebral artery diameter; VV, vertebral vasculitis.

### Diagnosis of GCA

**Figure 4:**
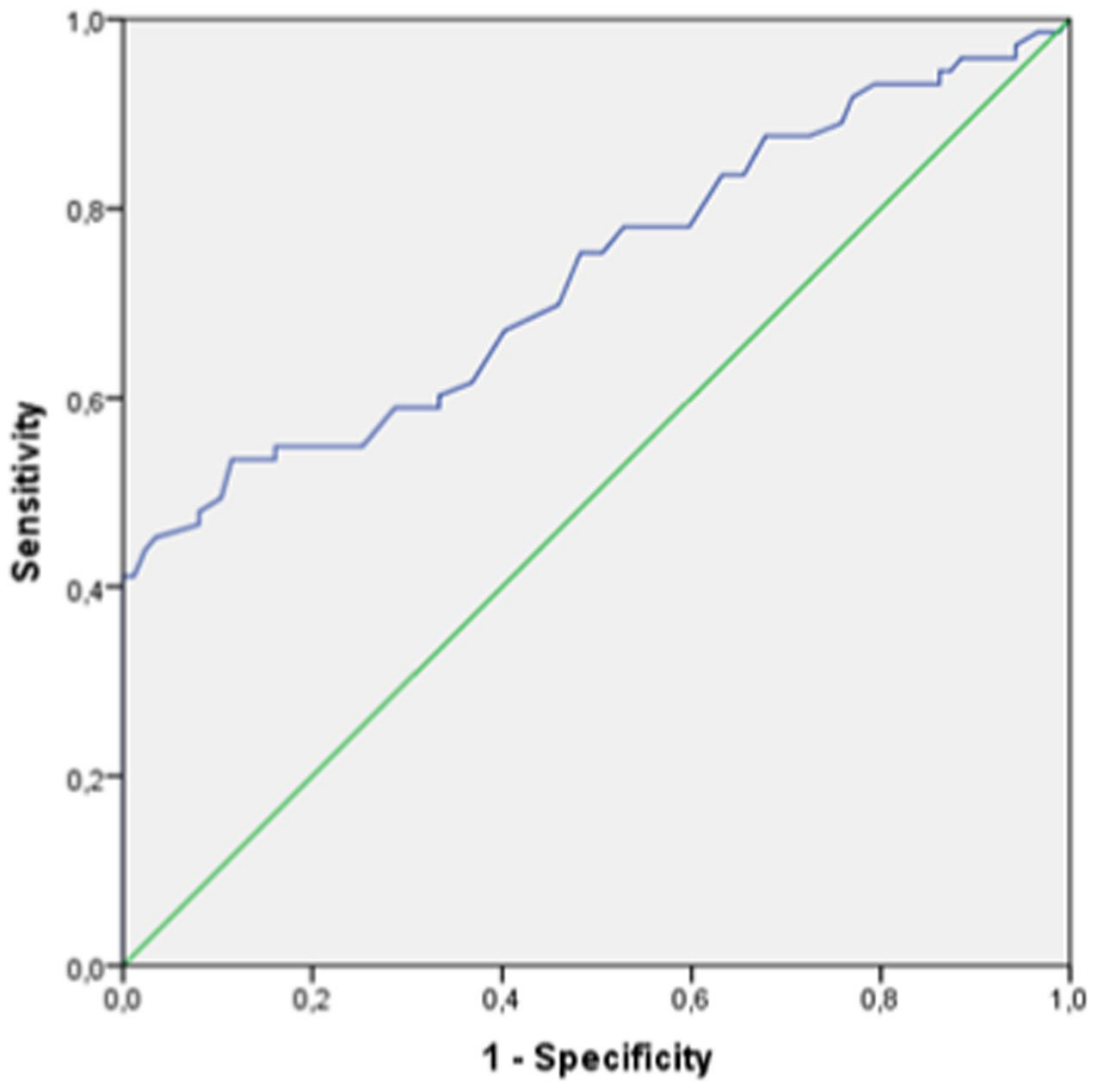
ROC curve for the evaluation of the validity of vertebral artery diameter to diagnose GCA.

The reliability values for the diagnosis of GCA by VAD was poor. In the student *t*-test study, the mean sum value of the vertebral diameter of both arteries was significantly higher in patients with GCA compared to controls without the disease (Table 7). However, the ROC curve had an area under the curve of 0.70, from which no diagnostic cut-off point values could be obtained (Figure 4).

**Table 7:** *t*-student test to compare the means of VAD diameter in patients with and without GCA.

	No GCA	GCA	*p*
Sum of VAD	6.9 (6.6–7.1)	8.5 (8.0–9.0)	<0.01

GCA, giant cell arteritis; VV, vertebral vasculitis; VAD, vertebral artery diameter.

### Evaluation of stroke risk

A student *t*-test study showed higher values of VAD in patients suffering an ischemic stroke in the vertebrobasilar area (Table 8).

**Table 8:** *t*-student test to compare the means of VAD diameter in patients with and without an ischemic stroke in the vertebrobasilar area.

	No stroke	Stroke	*p*
Sum of VAD (mm)	7.5 (7.2–7.7)	9.3 (8.3–10.5)	<0.01

GCA, giant cell arteritis; VAD, vertebral artery diameter.

In a multivariate study using logistic regression, VV was independently associated with the presence of stroke in the vertebrobasilar territory. The Sum of VAD was significantly associated with the presence of stroke. The risk of vertebro-basilar stroke increases 1.62 times in relation to the increase 1 mm in the Sum of VAD (Table 9).

**Table 9:** Multivariate analysis of factors associated to risk of ischemic stroke in the vertebrobasilar area.

	OR	*p*
Age	1.04 (0.96–1.12)	0.37
Sex	0.54 (0.16–1.88)	0.34
Sum of VAD	1.62 (1.19–2.19)	<0.01

GCA, giant cell arteritis; VAD, vertebral artery diameter.

## Discussion

In summary, the present study has demonstrated that in patients with GCA involving the cervical portion of the VA there is an increase in the caliber of the VAD detectable by ultrasonography. It has been possible to determine that the thickening is independent of other possible confounding factors.

Likewise, VAD has a high diagnostic reliability for VV in patients with GCA. The ideal way to evaluate the VAD can be defined as the sum of the diameter of the canal on both sides. This parameter shows an area under the curve with a very high value that corroborates the high diagnostic potential of the variable in an objective numerical form. Thus, it has been possible to establish cut-off values (Sum of VAD of 8.45 and 9.95 mm) with very high values of sensitivity, specificity, positive predictive value, and negative predictive value.

Notwithstanding, VAD, in isolation, shows low diagnostic reliability values for GCA.

Finally, the presence of thickening of the vertebral canal such as seen in this study is associated with the presence of an increased risk of ischemic stroke in the territory of the affected artery.

Regarding the main objective of the study, the VAD was clearly increased in those subjects who show vasculitic changes in the VA. To date, in our Laboratory of Neurosonology Laboratory, we only had the subjective impressions accumulated from the experience of physicians examining patients with GCA was evident. Now, all statistical analyses in this study have demonstrated this objectively by numerical analysis.

On the one hand, the results of the multivariate analysis have allowed us to determine that the association between VAD and the presence of vasculitis is independent of the cofactors studied. Given that this is a retrospective analysis, other variables that would have been interesting to include in the linear regression, such as the patient’s anthropometric indicators (height, weight, or body mass index), were unfortunately not available. Given the strong association between VAD and the existence of vasculitis, it is to be expected that these other variables were not a confounding factor explaining the association found. However, their inclusion would have given greater quality to the results presented.

The ROC curve analysis confirmed that the VAD variable with the greatest diagnostic value is the Sum of VAD. It is this variable that confer the maximum benefit for the diagnosis of VV.

As explained at the beginning of the results, it is common to find healthy subjects in whom, together with a hypoplastic VA, the contralateral VA has a somewhat larger diameter than usual. In daily clinical practice, the physician who performs studies of supra-aortic trunks is used to encountering this asymmetry. In the case of finding a VA with a small caliber flow signal, the physician must elucidate whether it is an individual constitutional characteristic or due to acquired pathology. One of the criteria that aid in determining that the small caliber artery is constitutional is to find the contralateral artery with a somewhat increased diameter, through which the flow is similar to that of subjects with symmetrical vertebral arteries (27, 29). Since physiological hyperplasia can reach values similar to those of arteries with vasculitis, the value that best defines such vasculitis is the Sum of VAD, where there is no contralateral hypoplasia in order to reduce the effect of constitutional confounder.

Consequently, when a study of supra-aortic trunks is performed by ultrasonography, it is part of the systematic study to check the caliber of the vertebral canal. Therefore, changes in VAD immediately draw attention. The knowledge of this diagnostic sign can help to have a suspicion of the presence of vasculitis just with the first B-mode image, even before checking other vasculitic changes by including the color mode.

Although vasculitic changes in the VA are usually detectable by sight for a physician accustomed to the study of this pathology, it is interesting to incorporate a new echographic sign that also provides numerical objective criteria, with robust validation. The reliability analysis indicates a very high discriminatory power of the variable, with a very low number of false positives and negatives. Specifically, in the case of the highest cut-off point (9.95 mm) there are no false positives at all, since all those with higher values show vasculitis in the VA. In addition, there are some patients in whom the assessment of the classic diagnostic criteria for VV is complicated (27). These criteria are based on the morphological evaluation of the lumen and the arterial wall, technically defined by the color mode. The VA is located in a deep area, and color mode differentiation may be poor, especially in patients with thick fat pads, somewhat limiting its utility. In the case of the VAD, the diameter data can be obtained in a simple way using only the B mode.

This echographic sign, the thickening of the VAD, is easy to recognize in any patient, even in those patients in whom there is no specific clinical suspicion of GCA when the study is performed. Its detection serves a dual purpose, not only detecting the presence of VV, but also completing the specific ultrasonographic study that leads to the diagnosis of GCA. Specifically, a high percentage of patients with GCA and VA involvement have a clinical presentation of ischemic stroke without the accompanying cardinal symptoms of GCA. In addition, the presence of vasculitis in this location is associated with a very poor clinical prognosis as seen in case of lack or delay of specific treatment (7, 11). For this reason, it is crucial to maximize efforts to detect this subgroup of patients with subtle symptoms of GCA but a higher risk of severe complications in the absence of proper diagnosis and specific treatment. Protocols for the etiological study of ischemic stroke usually incorporate ultrasonographic study of the supra-aortic trunks within the first few hours of hospital admission. This led to the recommendation of fast-track clinics for detecting GCA which includes extended ultrasound studies in the early phase of the disease (18, 30). This diagnostic approach has been associated with lower rates of complications and better outcome (3, 15). Knowing and detecting the thickening of the vertebral canal within VV due to GCA may be indirectly the key to reaching an etiological diagnosis, early enough to start specific treatment to avoid serious complications.

Despite the evidence of the usefulness of the use of ultrasound for the diagnosis of GCA, not all scientific societies recommend its use as the first diagnostic test (14). The contribution of new evidence on the use of the technique may help this recommendation to become unanimous.

With respect to the internal validity of this study, the methodology and the population studied allowed us to obtain results which constitute evidence for the defense of the initial hypothesis. Regarding external validity, the studied population includes, with a sufficiently large sample number, the three clinical categories studied in daily clinical practice. The proportions of subjects do not reflect the real prevalence of the disease in the general population. Therefore, the basal data provided are not valid for estimate the prevalence of the disease. Nevertheless, this is not part of the objects of the study. Instead, the selected population reinforces the study’s ability to evaluate the primary objective: the reliability of the vertebral diameter for the diagnosis of vasculitis in the vertebral artery. Specifically, it is worth emphasizing the size of the series of patients with GCA which was available for the study, including a high number of cases of VV. In general, the location studied, whilst possible, is reported as being infrequently used in many published clinical series (4). In addition, the studies were carried out with the required equipment/instruments in a laboratory as opposed to a clinical setting. Furthermore, the training required for the personnel performing the studies is the same as that required for any conventional supra-aortic trunk study.

There are no similar studies available that have analyzed the VAD as a sign of VV; this study is unprecedented to our knowledge and therefore the results cannot be compared with those of other studies. However, there are data available about the normal values of VAD in the normal population, which is comparable to the values of healthy subjects in this study (31).

In terms of evaluating the ability to diagnose the presence of giant cell arteritis by vertebral diameter, it does not seem to be a useful value, as indicated by the area under the ROC curve. However, we do see that all patients with pathological vertebral diameter values present with GCA. Therefore, although the vertebral diameter by itself is not a useful tool for the diagnosis of GCA, as we have previously stated, it has two advantages. On the one hand, in patients who are not believed to be suffering from GCA, the presence of vertebral vasculitis can facilitate the suspicion of this disease in patients without cardinal symptoms and the same ultrasonographic study can lead to the diagnosis of GCA by means of examination of the temporal artery. On the other hand, in those patients with known GCA, it is of great interest to discern whether the VA is affected, as this indicates a much higher risk of serious complications compared to those patients with unaffected vertebral arteries.

Among the study’s weaknesses, there are several issues worth discussing. Firstly, it has already been indicated that the study is retrospective. While this is generally a lower quality criterion for a study, given the results obtained in this study, it does not seem likely that a prospective study would reveal very much more. Secondly, it would have been interesting to have studies carried out by two different observers which would permit calculation of the K index. Since ultrasonography is postulated as a test in which the results may be observer-dependent, this would be valuable information. However, with this in mind, the measurement of the vertebral canal by ultrasonography is very simple and does not require specific equipment or software, nor special training, since it is part of a very basic procedure within ultrasonography. Therefore, significant interobserver variability is not expected.

Finally, it is, of course, worth highlighting the strengths of the study. It is important to emphasize the sample size of the work. It has been possible to collect clinical information and ultrasound studies of a very high number of patients with GCA, with and without VV. This has been the key to being confident in the ng able to defend the results presented as validity of the results.

## Conclusion

VAD as a marker of vasculitis in the VA by GCA is a highly sensitive and specific ultrasound sign, sufficiently reliable for use in routine clinical practice as a new diagnostic sign. As noted, it is easy to subjectively note the presence of an enlarged vertebral canal. The Sum of VAD leads the numerical variable with the best reliability to demonstrate VV. Given this, it is easy to locate an artery at risk of complications and even arouse suspicion of GCA in patients in whom there are no characteristic symptoms of this disease. Early diagnosis of GCA and VA involvement allows rapid initiation of pharmacological treatment, which has been shown to prevent complications, which are especially serious in the case of patients with vertebral involvement due to ischemic stroke in the territory of this artery (3, 11).

### Scope statement

The present study shows a new ultrasound sign with high reliability for diagnosing the presence of vertebral artery vasculitis in the context of giant cell arteritis. The vertebral diameter is very easy to measure for any person skilled in ultrasonography. In addition, the cut-off points provided clearly differentiate cases of GCA with vertebral vasculitis from the rest of subjects; both from healthy subjects and those with GCA without vertebral artery involvement. This marker improves the diagnostic benefits of ultrasonography for several reasons. Firstly, it facilitates the detection of the subgroup of patients with the highest risk of severe complications (ischemic stroke) and worse functional prognosis. Secondly, it may be the key to reach the diagnose of GCA in paucisymptomatic patients who present with ischemic stroke as the only clinical picture.

## Data availability statement

The original contributions presented in the study are included in the article/Supplementary material, further inquiries can be directed to the corresponding author.

## Ethics statement

The studies involving humans were approved by Comité de Ética de la Investigación con medicamentos (CEIm) by Gerencia de Atención Integrada de Albacete. The studies were conducted in accordance with the local legislation and institutional requirements. Written informed consent for participation was not required from the participants or the participants’ legal guardians/next of kin because The study was retrospective with an anonymous database.

## Author contributions

OA-M: Conceptualization, Formal analysis, Investigation, Methodology, Project administration, Resources, Supervision, Validation, Visualization, Writing – original draft, Writing – review & editing. JG-G: Conceptualization, Formal analysis, Investigation, Methodology, Resources, Validation, Visualization, Writing – review & editing. FH-F: Conceptualization, Formal analysis, Investigation, Methodology, Resources, Validation, Visualization, Writing – review & editing. MPo: Conceptualization, Formal analysis, Investigation, Methodology, Resources, Validation, Visualization, Writing – review & editing. BP-H: Investigation, Writing – review & editing. TB-E: Investigation, Writing – review & editing. AG-R: Investigation, Writing – review & editing. EM-C: Investigation, Writing – review & editing. BS-S: Conceptualization, Formal analysis, Investigation, Methodology, Resources, Validation, Visualization, Writing – review & editing. MPa: Conceptualization, Formal analysis, Investigation, Methodology, Validation, Visualization, Writing – review & editing. TS: Conceptualization, Formal analysis, Methodology, Project–administration, Resources, Validation, Visualization, Writing – review & editing.
