# Supplementary material for: Increased vertebral canal diameter measured by ultrasonography as a sign of vasculitis in patients with giant cell arteritis
Source: Front Med (Lausanne). 2023 Nov 7;10:1283285. doi: 10.3389/fmed.2023.1283285 (PMC10664249; doi:10.3389/fmed.2023.1283285)
Supplement: Supplementary file 1 [file Data_Sheet_1.PDF]

1. **ROC curve for calculating the reliability of vertebral diameter to diagnose vertebral vasculitis**

1.1. ROC curve for calculating the reliability of the vertebral diameter measured as " Unilateral VAD " to diagnose vertebral vasculitis

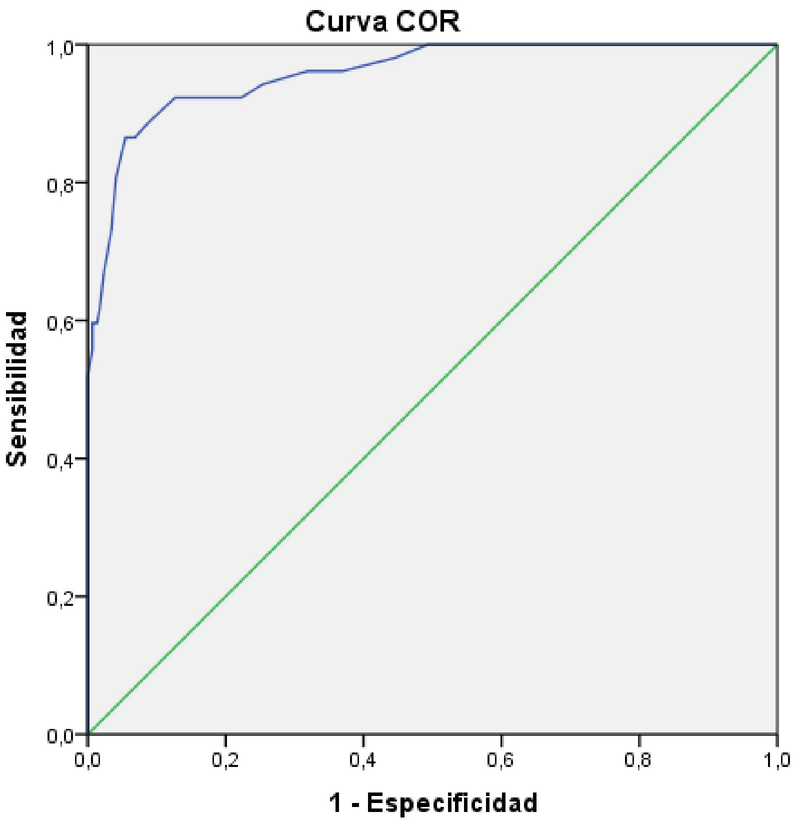

Los segmentos de diagonal se generan mediante empates.

Area down the curve

Test result variable(s): GLOBAL_DIAMETER

Area	standard error [a]	Asymptotic significance [b]	95% asymptotic confidence interval	
			Lower limit	Upper limit
.958	.014	,000	.931	.986

Curve coordinates

Test result variable(s): GLOBAL_DIAMETER

Positive if greater than or equal to	Sensitivity	1 - Specificity
.9000	1,000	1,000
1.9500	1,000	.993
2.0500	1,000	.979
2.1500	1,000	.973
2.2500	1,000	.969
2.3500	1,000	.959
2.4500	1,000	.949
2.5500	1,000	.942
2.6500	1,000	.914
2.7500	1,000	.870
2.8500	1,000	.836
2.9500	1,000	.812
3.0500	1,000	.771
3.1500	1,000	.733
3.2500	1,000	.651
3.3500	1,000	.589
3.4500	1,000	.555
3.5500	1,000	.493
3.6500	.981	.445
3.7500	.962	.370
3.8500	.962	,318
3.9500	.942	.253
4.0500	.923	,223
4.1500	.923	.175
4.2500	.923	.127
**4.3500**	**.885**	**.086**
4.4500	.865	.068
4.5500	.865	.055
4.6500	.808	.041
4.7500	.731	.034
4.8500	.673	.024
4.9500	.615	.017
5.0500	,596	.014
5.1500	,596	.007
5.2500	.558	.007
**5.3500**	**,519**	**,000**
5.4500	.481	,000
5.5500	.462	,000

5.6500	.404	,000
5.7500	.385	,000
5.8500	.346	,000
5.9500	.308	,000
6.0500	.288	,000
6,2000	,212	,000
6.4500	,192	,000
6.7500	.154	,000
7,0000	.058	,000
7.4500	.038	,000
7.9500	.019	,000
9,1000	,000	,000

1.2. COR curve prediction of the best cut-off point of the "Sum of diameters" variable to diagnose vertebral vasculitis

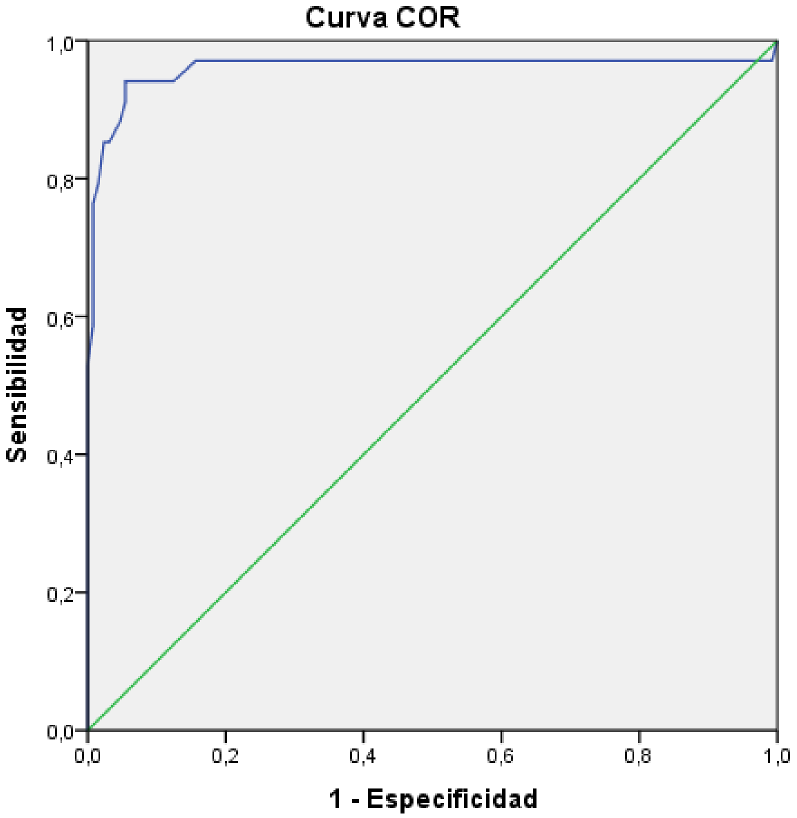

Los segmentos de diagonal se generan mediante empates.

Area down the curve

Test result variable(s): Sum of vertebral diameter

Area	standard error [a]	Asymptotic significance [b]
**.979**	.029	,000

Curve coordinates

Test result variable(s): Sum of vertebral diameter

Positive if greater than or equal to	Sensitivity	1 - Specificity
3.7000	1,000	1,000
4.7000	1,000	1,000
4.8000	.971	.992
4.9500	.971	.984
5,1000	.971	.977
5,2000	.971	.977
5.2500	.971	.953
5.3000	.971	.945
5.4000	.971	.922
5.5500	.971	.914
5.6500	.971	.906
5.7500	.971	.898
5.8500	.971	.891
5.9500	.971	.883
6.0500	.971	.836
6.1500	.971	.813
6,2000	.971	.813
6.2500	.971	.789
6.3000	.971	.766
6.3500	.971	.734
6.4500	.971	.695
6.5500	.971	.680
6.6500	.971	.625
6.7000	.971	.625
6.7500	.971	.578
6.8000	.971	.547
6.8500	.971	,531
6.9500	.971	.484
7.0500	.971	.430

7,1000	.971	.430
7.1500	.971	.367
7,2000	.971	.367
7.2500	.971	.336
7.3500	.971	.328
7.4500	.971	,313
7.5500	.971	.297
7.6500	.971	.258
7.7000	.971	.258
7.7500	.971	.227
7.8000	.971	,203
7.8500	.971	.195
7.9000	.971	.188
7.9500	.971	.164
8.0500	.971	.156
8.1500	.941	.125
8.2500	.941	.102
8.3500	.941	.094
8.4000	.941	.094
**8.4500**	**.941**	**.055**
8.6000	.912	.055
8.7500	.882	.047
8.9000	.853	.031
9,1000	.853	.023
9.2500	.794	.016
9.4000	.765	.008
9.5500	.706	.008
9.6000	.676	.008
9.7000	.647	.008
9.8500	.588	.008
**9.9500**	**,529**	**,000**
10.1500	.471	,000
10.3500	.412	,000
10.4500	.382	,000
10.5500	.324	,000
10.8000	.265	,000
11.1500	.235	,000
11,4000	.176	,000
11.7500	.147	,000
12.2500	,118	,000
13.1500	.088	,000
14,1000	.029	,000
15,4000	,000	,000

2. **Cross tables to calculate Sensitivity, Specificity, Positive Predictive Value and Negative Predictive Value with each of the two cut-off points**

2.1. **Cross tables to calculate Sensitivity, Specificity, Positive Predictive Value and Negative Predictive Value with each of the two cut-off points using the "Unilateral VAD" variable.**

2.1.1. **For the cutting point 4.35mm**

Cross table

			VERTEBRAL VASCULITIS		
			NO VASCULITIS	VASCULITIS	Total
diameter dichotomized at 4.35mm	<8.45mm	Count	267	6	273
		% within Global diameter dichotomized at 4.35	97.8%	2.2%	100.0%
		% within global vertebral halo	**91.4%**	11.5%	79.4%
	≥8.45mm	Count	25	46	71
		% within Global diameter dichotomized at 4.35	35.2%	64.8%	100.0%
		% within global vertebral halo	8.6%	**88.5%**	20.6%
Total		Count	292	52	344
		% within Global diameter dichotomized at 4.35	84.9%	15.1%	100.0%
		% within global vertebral halo	100.0%	100.0%	100.0%

Chi-square tests

	Worth	gl	Asymptotic sig. (2-sided)	Exact meaning (2 sides)	Exact meaning (1 side)
Pearson chi-square	172,035 [a]	1	,000		
Continuity correction [b]	167,191	1	,000		
Likelihood ratio	142,406	1	,000		
Fisher's exact test				,000	,000
Linear-by-linear association	171,535	1	,000		
No. of valid cases	344				

2.2. Overall diameter dichotomized at 5.35 mm

Cross table

			VERTEBRAL VASCULITIS		Total
			NO VASCULITIS	VASCULITIS	
diameter dichotomized at 5.35mm	<5.35	Count	292	25	317
		% within Global diameter dichotomized at 5.35	92.1%	7.9%	100.0%
		% within global vertebral halo	100.0%	48.1%	92.2%
	≥5.35mm	Count	0	27	27
		% within Global diameter dichotomized at 5.35	0.0%	100.0%	100.0%
		% within global vertebral halo	0.0%	51.9%	7.8%
Total		Count	292	52	344
		% within Global diameter dichotomized at 5.35	84.9%	15.1%	100.0%
		% within global vertebral halo	100.0%	100.0%	100.0%

Chi-square tests

	Worth	gl	Asymptotic sig. (2-sided)	Exact meaning (2 sides)	Exact meaning (1 side)
Pearson chi-square	164,529 [a]	1	,000		
Continuity correction [b]	157,428	1	,000		
Likelihood ratio	117,232	1	,000		
Fisher's exact test				,000	,000
Linear-by-linear association	164,051	1	,000		
No. of valid cases	344				

to. 1 cells (25.0%) have expected a count less than 5. The minimum expected count is 4.08.
b. It has only been calculated for a 2x2 table

2.3. Cross tables to calculate Sensitivity, Specificity, Positive Predictive Value and Negative Predictive Value with each of the two cut-off points using the variable "Sum of VAD").

2.3.1. with the cutting point at 8.45 mm:

Vertebral sum section 8.45*vertebral arteritis crosstabulation

			vertebral vasculitis		Total
			NO	YEAH	
sum cut 8.45mm	< 8.45mm	Count	121	2	123
		% within Sum vertebral cut 8.45	**98.4%**	1.6%	100%
		% within vertebral arteritis	**94.5%**	5.9%	
		% of the total	74.7%	1.2%	75.9%
	≥8.45mm	Count	7	32	39
		% within Sum vertebral cut 8.45	17.9%	**82.1%**	**100%**
		% within vertebral arteritis	5.5%	**94.1%**	
		% of the total	4.3%	19.8%	24.1%
Total		Count	128	3. 4	162
		% within Sum vertebral cut 8.45	79.0%	21.0%	
		% within vertebral arteritis	100.0%	100.0%	100%
		% of the total	79.0%	21.0%	

Chi-square tests

	Worth	gl	Asymptotic sig. (2-sided)	Exact meaning (2 sides)	Exact meaning (1 side)
Pearson chi-square	115,500 to	1	,000		
Continuity correction b	110,701	1	,000		
Likelihood ratio	109,318	1	,000		
Fisher's exact test				,000	,000
Linear-by-linear association	114,787	1	,000		
No. of valid cases	162				

to. 0 cells (.0%) have expected a count less than 5. The minimum expected count is 8.19.
b. It has only been calculated for a 2x2 table

2.3.2. sum cut 9.95*vertebral arteritis cross tabulation **2.3.3**

			vertebral arteritis		Total
			NO	YEAH	
sum cut 9.95mm	<9.95mm	Count	128	16	144
		% within Sum vertebral cut 9.95	**88.9%**	11.1%	100%
		% within vertebral arteritis	**100.0%**	47.1%	
		% of the total	79.0%	9.9%	88.9%
	≥9.96mm	Count	0	18	18
		% within Sum vertebral cut 9.95	0.0%	**100.0%**	100%
		% within vertebral arteritis	0.0%	**52.9%**	
		% of the total	0.0%	11.1%	11.1%
Total		Count	128	3. 4	162
		% within Sum vertebral cut 9.95	79.0%	21.0%	
		% within vertebral arteritis	100.0%	100.0%	
		% of the total	79.0%	21.0%	

Chi-square tests

	Worth	gl	Asymptotic sig. (2-sided)	Exact meaning (2 sides)	Exact meaning (1 side)
Pearson chi-square	76,235 [a]	1	,000		
Continuity correction [b]	70,969	1	,000		
Likelihood ratio	66,005	1	,000		
Fisher's exact test				,000	,000
Linear-by-linear association	75,765	1	,000		
No. of valid cases	162				
