# Supplementary material for: Increased vertebral canal diameter measured by ultrasonography as a sign of vasculitis in patients with giant cell arteritis
Source: Front Med (Lausanne). 2023 Nov 7;10:1283285. doi: 10.3389/fmed.2023.1283285 (PMC10664249; doi:10.3389/fmed.2023.1283285)
Supplement: Supplementary file 2 [file Data_Sheet_2.PDF]

1. **MULTIVARIATE STUDY USING LOGISTIC REGRESSION TO KNOW THE VARIABLES THAT ARE INDEPENDENTLY ASSOCIATED WITH THE PRESENCE OF VERTEBRAL VASCULITIS,**

Logistic regression

Variables in the equation

		b	Standard error	Wald	gl	Next.	Exp (B)
Step 0	Constant	-1,302	,193	45,309	1	,000	.272

The variables are not in the equation

			Punctuation	gl	Next.
Step 0	Variables	AGE	9,831	1	.002
		SEX	.102	1	.749
		SUM OF VAD	88,613	1	,000
	Global statistics		89,783	3	,000

Block 1: Method = Advance by step (Conditional)

Model Summary

Step	Log likelihood -2	Cox and Snell R-squared	**Nagelkerke R-squared**
1	57,803 [a]	.491	**.760**

to. The estimation has ended at iteration number 7 because the parameter estimates have changed by less than .001.

Variables in the equation,

		b	Standard error	Wald	gl	Next.
Step 1 [a]	**VERTEBRALS_SUM**	2,091	.377	30,799	1	**,000**
	Constant	-18,938	3,303	32,879	1	,000

Variables in the equation,

		Exp (B)	95% CI for EXP(B)	
			lower	Superior
Step 1 [a]	**VERTEBRALS_SUM**	**8,091**	**3,867**	**16,931**
	Constant	,000		

to. Variables specified in step 1: VERTEBRALES_SUM.

The variables are not in the equation

			Punctuation	gl	Next.
Step 1	Variables	AGE	,899	1	**.343**
		SEX	2,249	1	**.134**
	Global statistics		3,404	2	.182
